# Supplementary material for: Exploiting a Y chromosome-linked Cas9 for sex selection and gene drive
Source: Nat Commun. 2021 Dec 10;12:7202. doi: 10.1038/s41467-021-27333-1 (PMC8664916; doi:10.1038/s41467-021-27333-1)
Supplement: Supplementary file 1 — Supplementary Information [file 41467_2021_27333_MOESM1_ESM.pdf]

Supplementary Information for Exploiting a Y Chromosome linked Cas9 for Sex Selection and Gene drive

Author list:
Stephanie Gamez[1,10,#], Duverney Chaverra-Rodriguez[1,#], Anna Buchman[1,11], Nikolay P. Kandul[1], Stelia C. Mendez- Sanchez[1,2], Jared B. Bennett[3,4], Hector M. Sanchez C.[4], Ting Yang[1], Igor Antoshechkin[5] , Jonny E. Duque[1,6], Philippos A. Papathanos[7], John Marshall[4,9], Omar S. Akbari[1,†].

Affiliations:
[1] Division of Biological Sciences, Section of Cell and Developmental Biology, University of California, San Diego, La Jolla, CA 92093, USA
[2] Group for Research in Biochemistry and Microbiology (Grupo de Investigación en Bioquímica Y Microbiología-GIBIM), School of Chemistry, Universidad Industrial de Santander, Bucaramanga, Colombia
[3] Biophysics Graduate Group, University of California, Berkeley, CA, 94720, USA
[4] Divisions of Epidemiology & Biostatistics, School of Public Health, University of California, Berkeley, CA 94720, USA
[5] Division of Biology and Biological Engineering (BBE), California Institute of Technology, Pasadena, CA 91125
[6] Centro de Investigaciones en Enfermedades Tropicales – CINTROP, Facultad de Salud, Escuela de Medicina, Departamento de Ciencias Básicas, Universidad Industrial de Santander, Piedecuesta, Santander, Colombia
[7] Department of Entomology, Robert H. Smith Faculty of Agriculture, Food and Environment, Hebrew University of Jerusalem, Rehovot 7610001, Israel
[9]Innovative Genomics Institute, University of California, Berkeley, CA 94720, USA
[10]Present address: Agragene Inc., San Diego, CA 92121, USA
[11]Present address: Verily Life Sciences, South San Francisco, CA 94080, USA

[#] Equal contributions

[†]To whom correspondence should be addressed:
Omar S. Akbari, Ph.D.; Division of Biological Sciences, Section of Cell and Developmental Biology, University of California, San Diego, La Jolla, CA 92093, USA; Ph: 858-246-0640; Email: oakbari@ucsd.edu

a

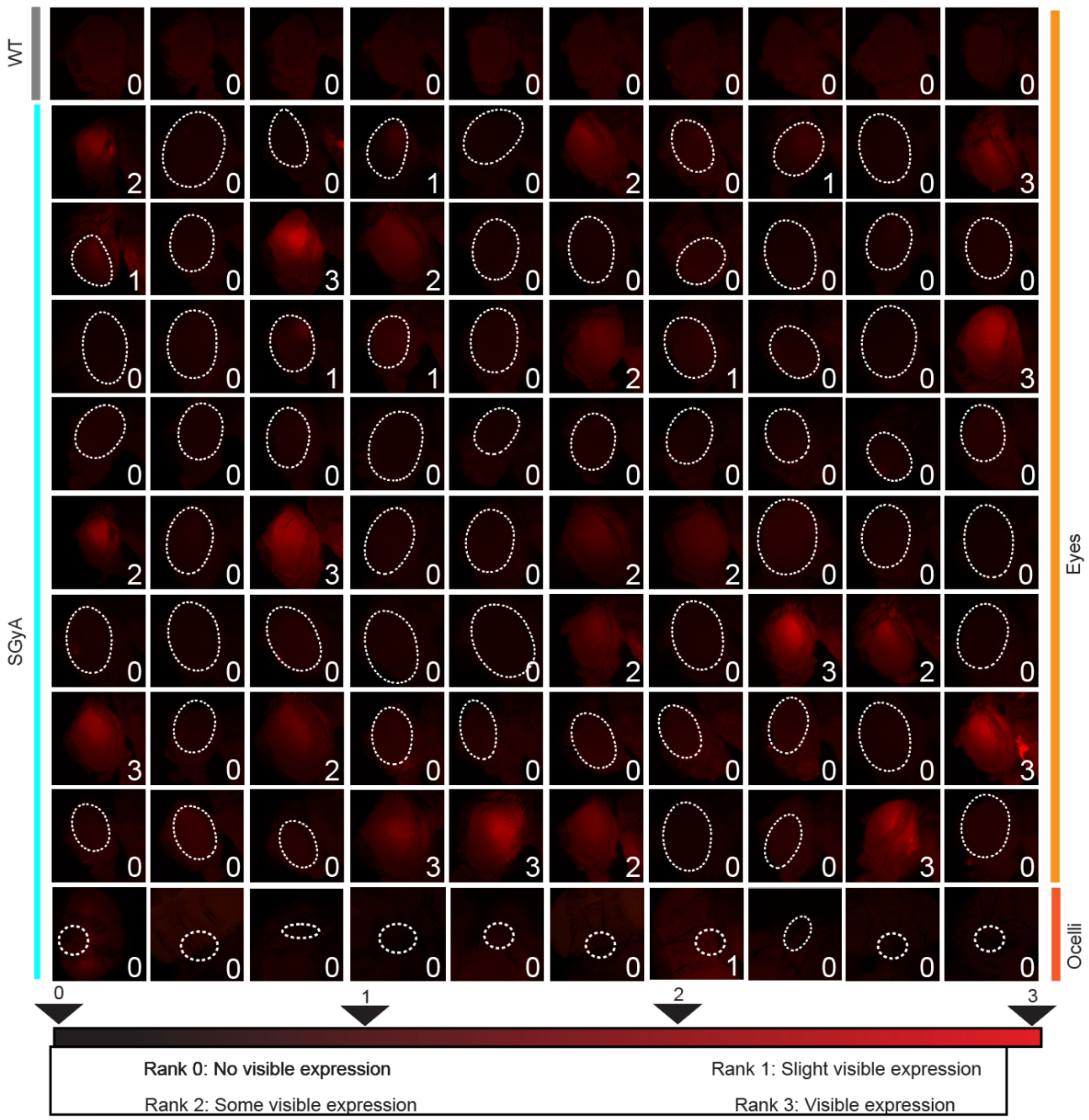

WT
SGyA
Eyes
Ocelli
0
1
2
3
Rank 0: No visible expression
Rank 1: Slight visible expression
Rank 2: Some visible expression
Rank 3: Visible expression

b

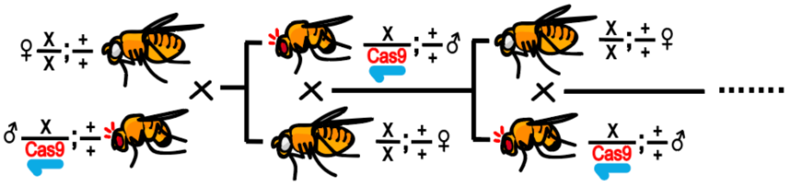

Cas9

c

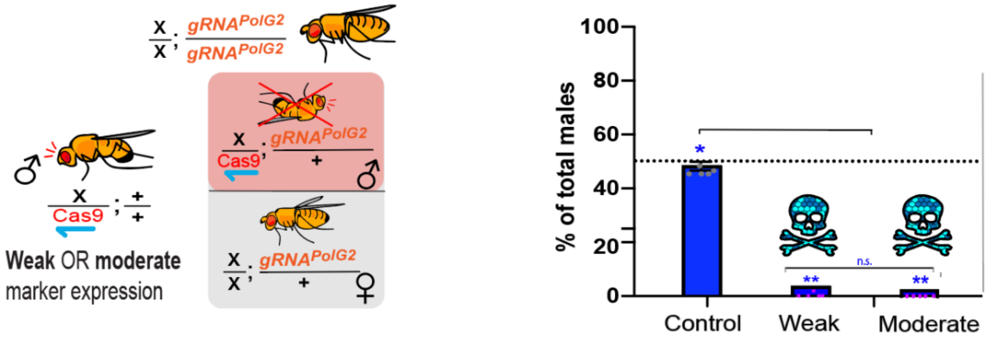

gRNA^PolG2
Cas9
Weak OR moderate
marker expression
% of total males
100
80
60
40
20
0
n.s.
Control
Weak
Moderate

Supplementary Figure 1. Variability of fluorescent marker expression in SGyA males. (A) Due to the heterochromatic nature of the Y chromosome, the SGyA transgene marker shows variability in expression. In 80 randomly sampled transgenic males, the major proportion of these flies had little to no visible marker expression. Each image was ranked with a number between 0-3. Numbers indicated in bottom right corners indicate the ranking of marker expression where 0 is no visible expression and 3 represents visible marker expression. Confirmation PCRs show that the transgene is present in their genomes, despite the lack of a strong visible marker expression. Ocelli images of 10 SGyA flies were taken to show variability in their expression. WT fly images were taken (first top row) to compare control flies to transgenic flies. **(B)** Mating schematic of how SGyA line is maintained. In the initial cross set up, SGyA males were outcrossed to WT females. Progeny of this mating produced SGyA males and WT females. These siblings are allowed to mate and propagate the transmission of the SGyA transgene through males. (**C)** In order to determine if marker expression was associated with Cas9 functionality, males with weak (Rank 0-1) or moderate (Rank 2-3) expression of the SGyA marker were crossed to homozygous females carrying a sgRNA targeting *PolG2.* Knockout of this gene produces lethal biallelic mosaicism that causes the death of affected progeny. We observed significant differences in male survival between the progeny from transgenic males and the wildtype control (derived from the cross of wildtype males crossed to homozygous gRNA females), but we found no difference in male killing effects on the progeny derived from weak or moderate marker expression males. For cross experiments, at least 6 replicate crosses were performed. Significance was determined using a two-tailed student's t-test. Vertical bars represent SEM. n.s. represents Non-significant. *$p < 0.001$; **$p = 0.3466$. Source data is provided as a Source Data file.

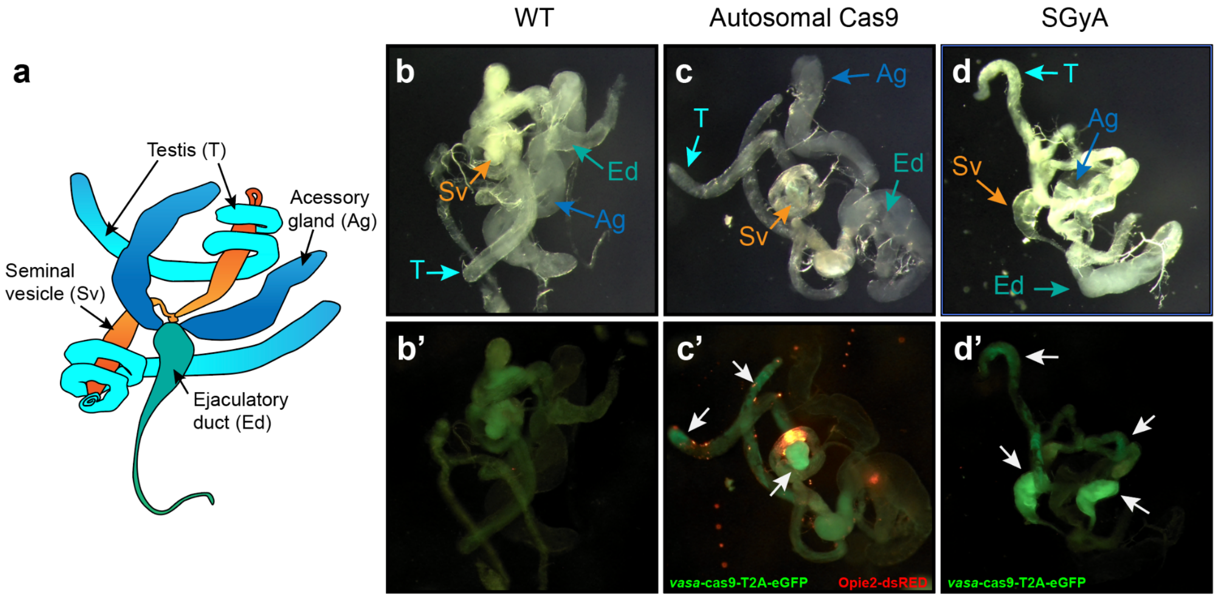

Supplementary Figure 2. *vasa*-T2A-eGFP expression of dissected testes from WT, autosomal-Cas9, and SGyA males. (A) Schematic depicting testes anatomy. Each major structure is highlighted, testis (T; in teal), accessory gland (Ag; in dark blue), seminal vesicle (Sv; in orange), and ejaculatory duct (Ed; in blue-green). **(B-D)** images of dissected testes in white light. **(B'-D')** fluorescent images of dissected testes under a GFP/RFP filter. (E) No fluorescence is observed in the WT testes due to lack of T2A-eGFP. **(C')** Autosomal-Cas9 testes demonstrate eGFP expression in the testes and seminal vesicles. In addition, they also exhibit some dsRED expression (reflecting the Opie2-dsRED marker). **(D')** Visible eGFP expression in SGyA testes and seminal vesicles. Similar levels of eGFP expression are apparent in both autosomal-Cas9 and SGyA samples. In the white light images, color coded arrows (corresponding to panel A), indicate the location of testes structures. White arrows indicate points where eGFP is visible in the fluorescent images. Fluorescent and non fluorescent images were consistently observed throughout the screening of sampled lines (n < 5).

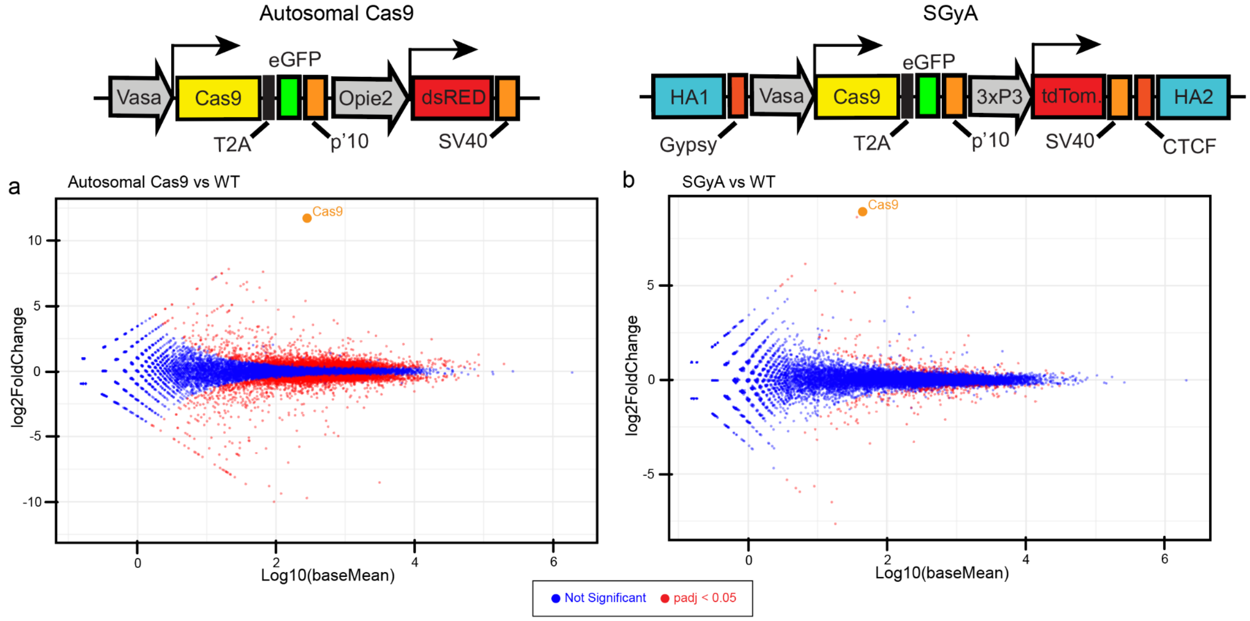

Supplementary Figure 3. Expression analyses of gene expression levels (indicated by TPM [transcripts per million] values) in autosomal Cas9, SGyA and wildtype flies. Total RNA from adult male flies aged 3-4 days were extracted and sent for RNA-sequencing to obtain quantitative estimates of expression levels. **(A-B)** MA plots depicting expression of genes in autosomal-Cas9 vs WT in **(A)** and SGyA vs WT in **(B)**. Cas9 expression is significant in transgenic samples compared to WT. log2FoldChange of Cas9 in SGyA is lower compared to that of autosomal Cas9, demonstrating distinct levels of expression. The y-axis represents the log2FoldChange and the x-axis represents the log10 (baseMean). The top of the plots indicate genes that are highly expressed in transgenic flies while genes found below 0 correspond to genes highly expressed in WT flies. Points in red are significant at *padj*<0.05. Larger point sizes in panels A-B are to increase visibility. Corresponding DeSeq results are located in **Table S2** (for autosomal vs WT) and **Table S3** (for SGyA vs WT).

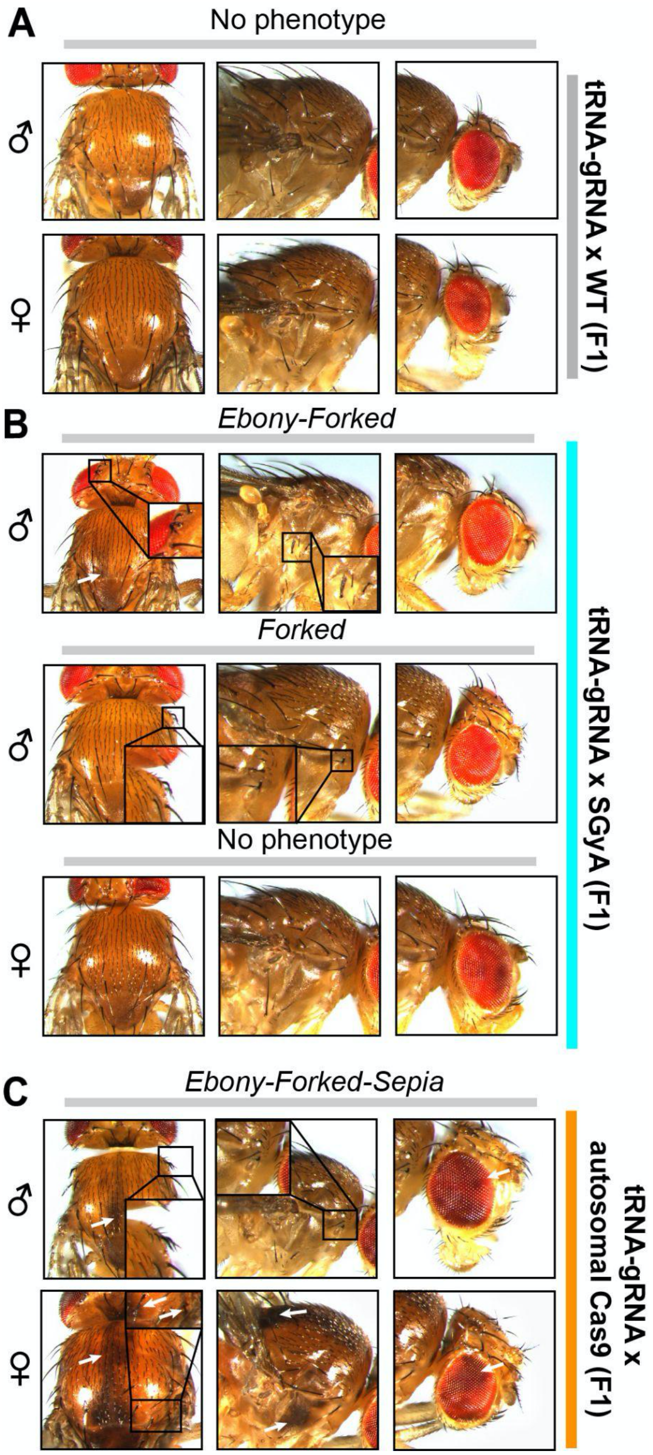

A
No phenotype
♂
♀
tRNA-gRNA x WT (F1)
B
Ebony-Forked
Forked
No phenotype
tRNA-gRNA x SGyA (F1)
C
Ebony-Forked-Sepia
tRNA-gRNA x autosomal Cas9 (F1)

Supplementary Figure 4. Sex-bias of mutant phenotypes observed in F1 progeny from crosses with a control (WT), SGyA, or an autosomal source of Cas9. The data depicts the percentages of phenotypes present in all the males (or females) scored. In SGyA crosses, only F1 males demonstrated mutant phenotypes compared to the non-mutant female progeny. No sex-bias of mutations in F1 progeny from autosomal Cas9 crosses. Blue bars represent the percentage of males and pink represents the percentage of females with particular mutant phenotypes. Please refer to **Table 1** for ANOVA comparisons of the percentages of mutant phenotypes among F1 progeny from each experimental group. **(A-C)** Selection of phenotypes of mutations in F1 progeny from crosses involving SGyA, autosomal Cas9, or the control (multiplexed gRNA strain). **(A)** Control crosses did not exhibit any mutant F1 progeny due to lack of Cas9. **(B)** Mutant phenotypes in only male progeny from SGyA crosses are subtle and range in severity. females did not exhibit mutant phenotypes. **(C)** Mutant phenotypes from autosomal Cas9 crosses are stronger and easier to distinguish. Both males and females were affected and often displayed a triple somatic phenotype. White arrows indicate somatic mutation phenotypes.

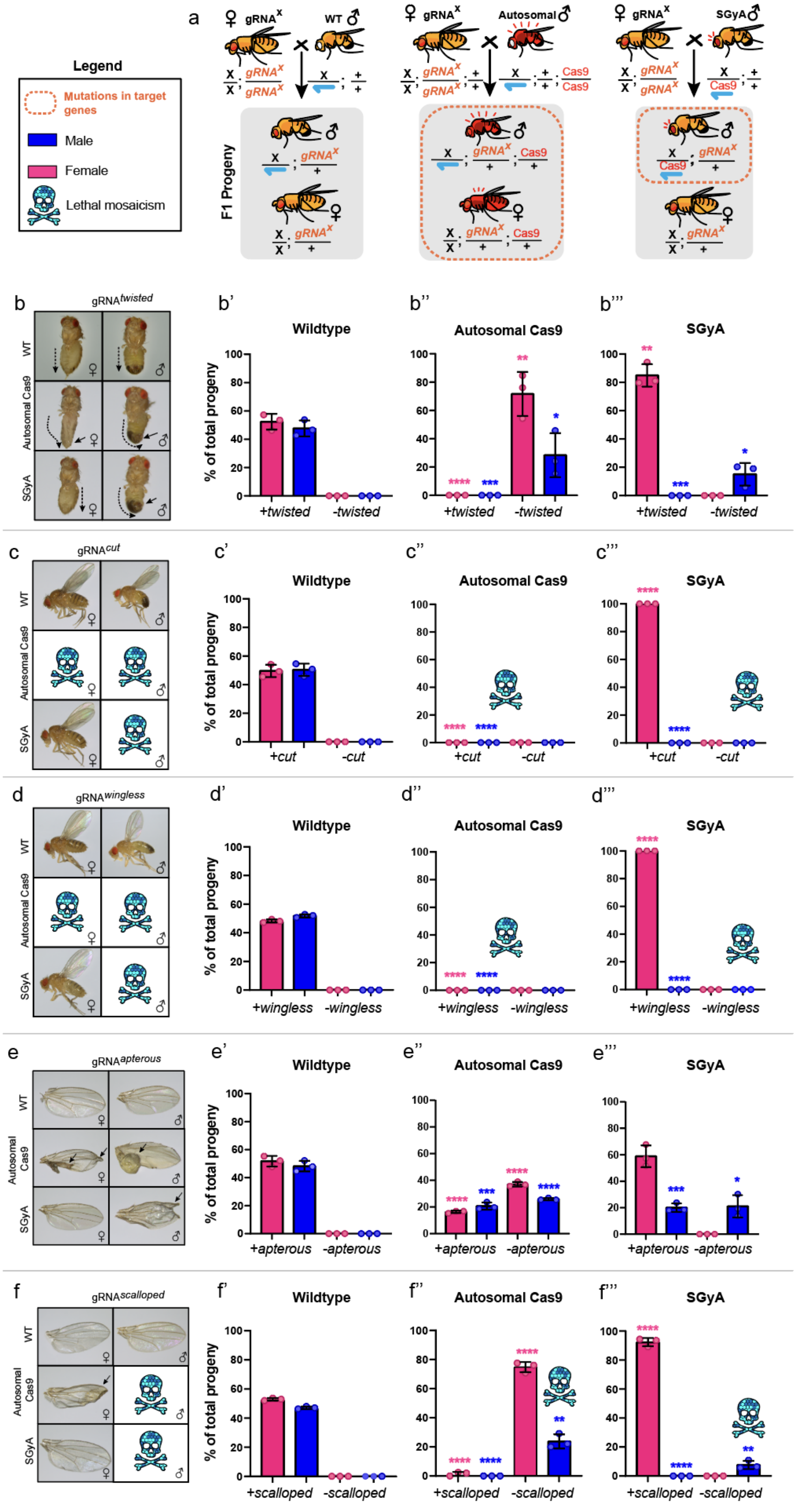
Legend
Mutations in target genes
Male
Female
Lethal mosaicism
a
F1 Progeny
b
b'
b''
b'''
Wildtype
Autosomal Cas9
SGyA
% of total progeny
+twisted
-twisted
c
c'
c''
c'''
+cut
-cut
d
d'
d''
d'''
+wingless
-wingless
e
e'
e''
e'''
+apterous
-apterous
f
f'
f''
f'''
+scalloped
-scalloped

Supplementary Figure 5. Mutant phenotypes of five gRNA target sites in F1 progeny with different sources of Cas9. (A) Cross schematic of control and experimental crosses performed using gRNA^{x} (where X represents one of the five phenotypic genes targeted). The five phenotypic target sites, **(B)** *twisted*, **(C)** *cut*, **(D)** *wingless*, **(E)** *apterous*, and **(F)** *scalloped* were tested against WT, SGyA, and an autosomal source of Cas9. As expected, no mutant phenotypes were seen in WT crosses. When gRNAs are outcrossed to SGyA, mutant phenotypes are only seen in males. Females from these crosses were unaffected. In crosses with autosomal Cas9, both males and females were affected. **(B)** Twisted abdomens are seen in both F1 male and female progeny of autosomal sources of Cas9 whereas only males are affected in Y-linked Cas9 crosses. Quantitative data for F1 progeny outcomes for WT, autosomal Cas9, and SGyA crosses are plotted in B', B'', and B''', respectively. Early embryo lethality was seen in crosses involving **(C, C', C'', and C''')** cut and late pupal lethality was seen in **(D, D', D'', and D''')** wingless. For **(E)**, autosomal Cas9 crosses produced wing deformities in males and females. For **(F, F', F'', and F''')**, F1 progeny who inherited the gRNA were mostly lethal (especially in males; no deformed wings shown). Interestingly, all F1 females from the autosomal Cas9 crosses survived, however, maintained deformed wings. Individuals with '+gene' genotypes indicate presence of the gRNA and a wildtype target site. Individuals with '-gene' genotypes indicate the presence of the gRNA and a mutant phenotype of the associated target site. Three replicates were set up per experimental cross. A two-tailed student's t-test was used to determine significance of percentages compared to respective WT categories. Error bars in black represent the mean and +/- SEM. ****$p < 0.0001$; ***$p < 0.001$; **$p < 0.005$; *$p < 0.05$. Source data is provided as a Source Data file.

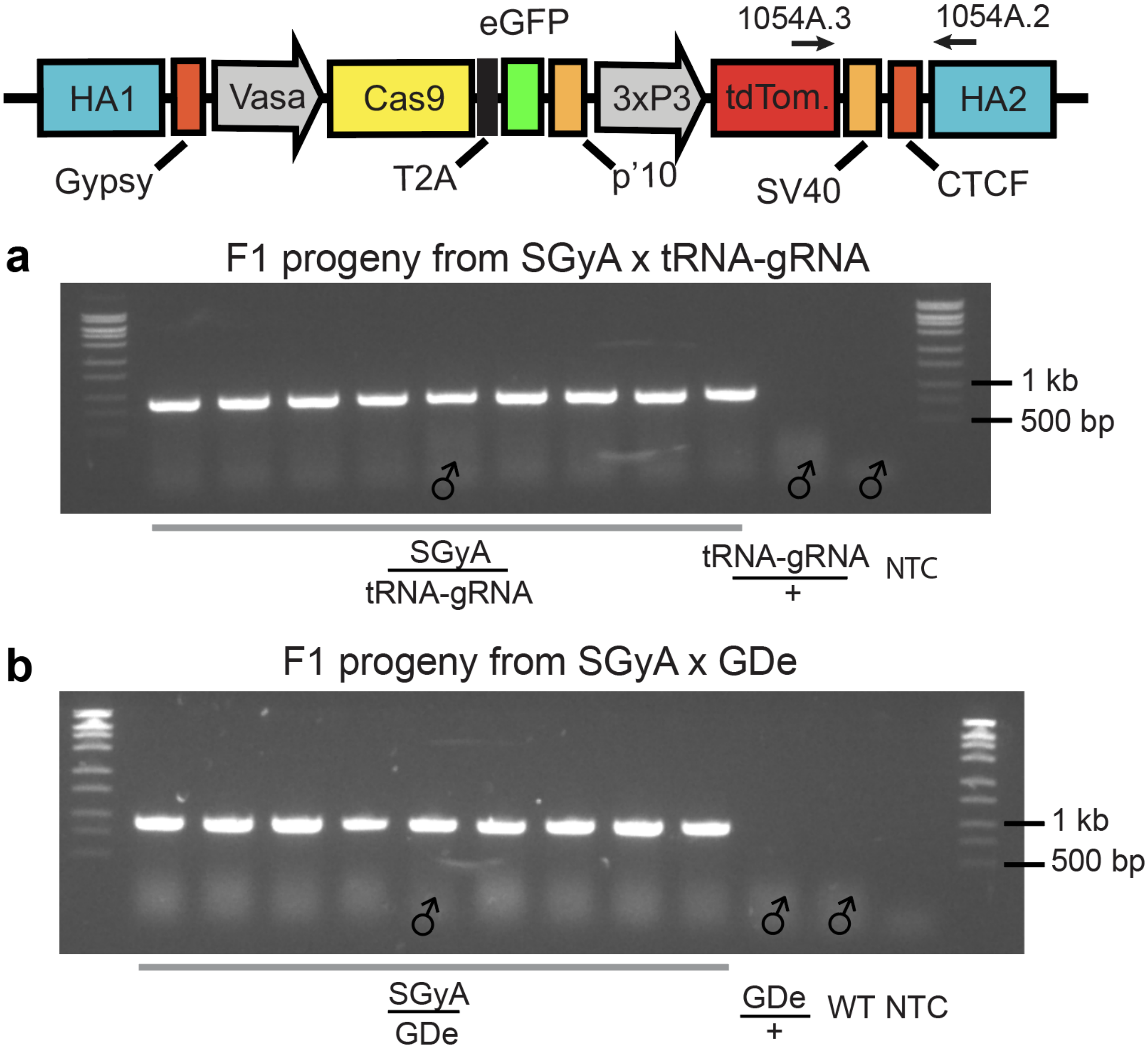

Supplementary Figure 6. PCR confirmation of F1 males carrying the SGyA transgene. SGyA transgenes were confirmed in F1 males from crosses involving SGyA against the multiplexed tRNA-gRNA and GDe lines. Fluorescent markers for the SGyA transgene were difficult to observe under a microscope due to their low expression pattern from the Y chromosome. PCRs were carried out with primers 1054A.3 and 1054A.2 to confirm the presence of the SGyA transgene in 9 random F1 males. **(A)** F1 transheterozygous males from SGyA x tRNA-gRNA crosses inherited the SGyA transgene. **(B)** F1 transheterozygous males from SGyA x GDe crosses inherited the SGyA transgene. Amplification was performed at least twice by two independent scientists.

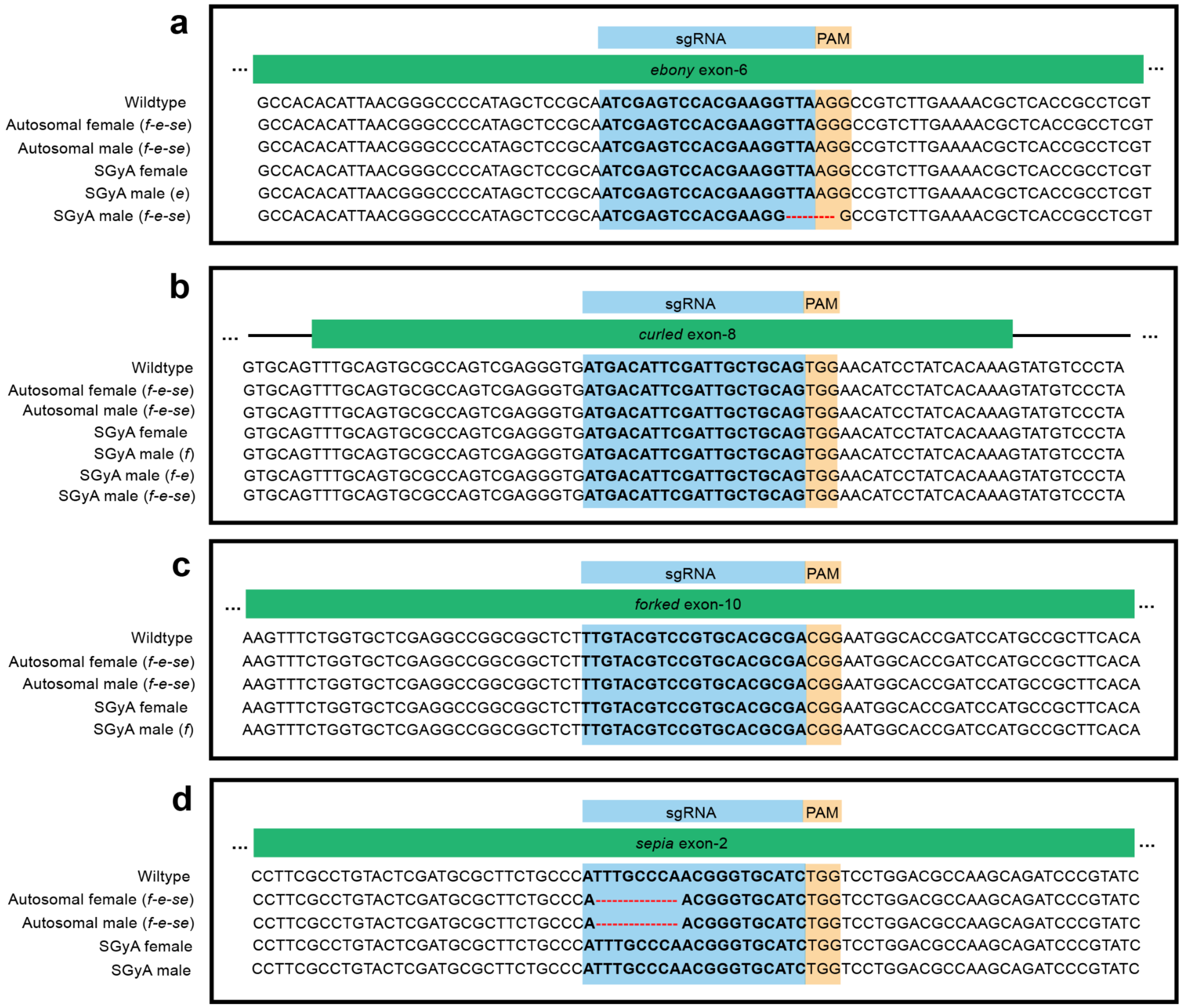

Supplementary Figure 7. Sequencing results of four target sites in F1 progeny generated by a multiplexed tRNA-gRNA system and different sources of Cas9. The multiplexed tRNA-gRNA targets four genes, **(A)** *ebony*, **(B)** *curled*, **(C)** *forked*, and **(D)** *sepia*. To assess mutations, F1 progeny from crosses between the tRNA-gRNA multiplexed system and different sources of Cas9 (autosomal or Y-linked) were examined. The sgRNA target site is highlighted in blue and the PAM sequence in yellow. The exon portion of each gene is colored in green and the intron is indicated in a black line. The target site sequence is in bold. Mutations are indicated in red. Individuals with mutations are indicated in parentheses. For example, in panel **(B)**, the sequenced "SGyA male (*f-e-se*)" had a triple mutant phenotype of *forked, ebony* and *sepia*. Those without parentheses did not have any visible mutations. For the majority of mutant individuals sequenced, no mutations were seen at the target site **(A-D)**. A five base pair deletion was detected in a male triple mutant from the SGyA cross **(A)**. Deletions were observed in sepia mutants from autosomal Cas9 crosses **(D)**.

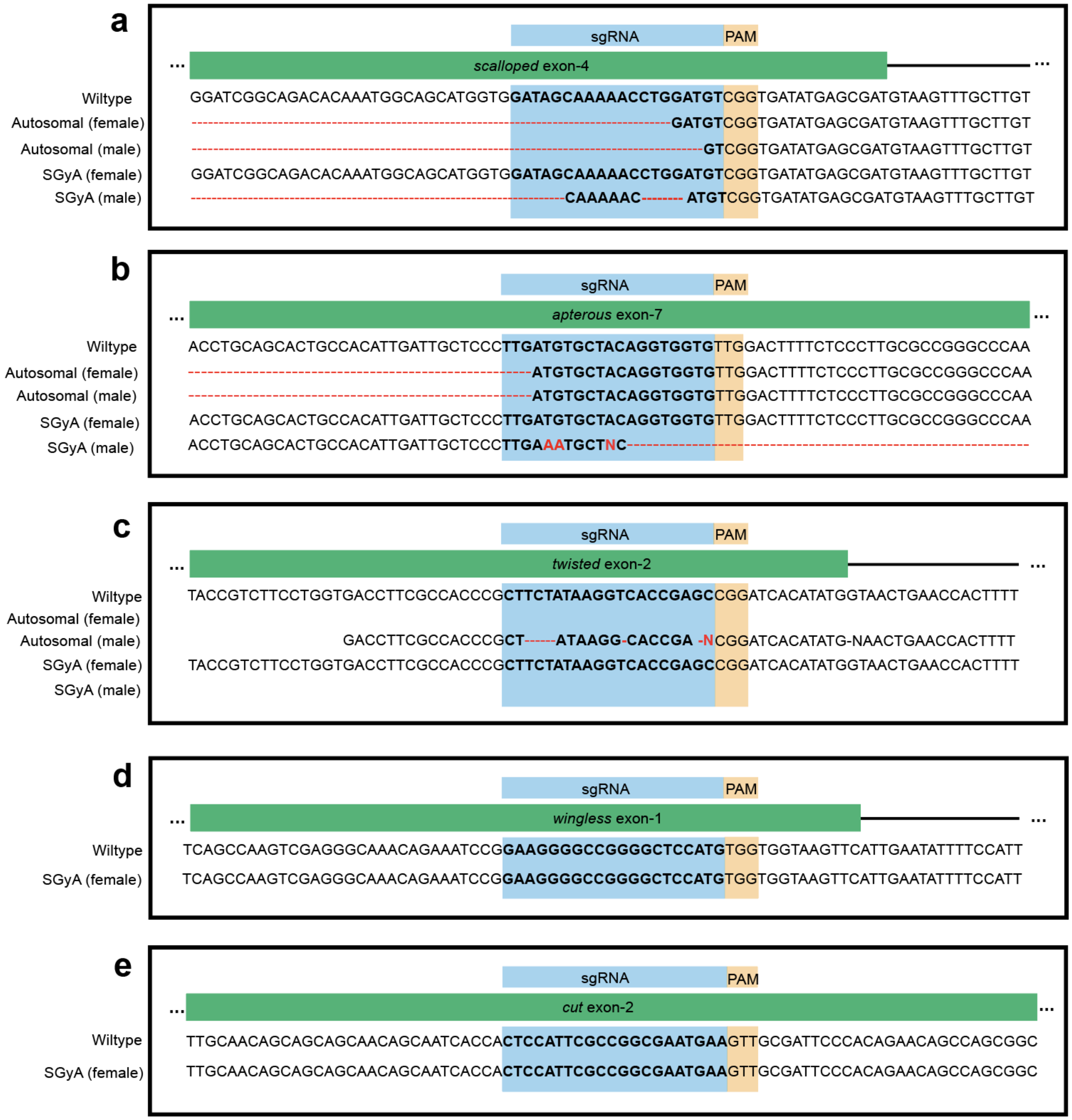

Supplementary Figure 8. Sequencing results of five sgRNA target sites in F1 progeny with different sources of Cas9. Target sites for each gRNA tested **(A)** *scalloped*, **(B)** *apterous*, **(C)** *twisted*, **(D)** *wingless*, **(E)** *cut* were PCR amplified and sequenced to assess the mutations of F1 progeny in crosses involving autosomal-Cas9 and a Y-linked source of Cas9. The sgRNA target site is highlighted in blue and the PAM sequence in yellow. The exon portion of each gene is colored in green and the intron is indicated in a black line. The target site sequence is in bold. Mutations are indicated in red. A single F1 female or male from each experimental cross was sequenced with the exception of males and females from autosomal crosses with *wingless* and *cut* sgRNAs and males from *wingless* and *cut* crosses. As expected, no mutations were observed in SGyA females when crossed to any of the five sgRNAs. All males and females are affected in

crosses involving autosomal-Cas9 and result in mutations at the target site that lead to mutant phenotypes or lethality.

Supplementary Data File 1. Quantification of total RNA expression in WT, autosomal-Cas9, and SGyA adult ♂ whole body at 3-4 days old. Both raw read counts and normalized TPM values are included.
Supplementary Data File 2. Two-factor comparison between autosomal Cas9 and WT.
Supplementary Data File 3. Two-factor comparison between SGyA and WT.
Supplementary Data File 4. List of model and intervention parameters.

Supplementary Table 1. Primer sequences used in this study

Primer name	Primer Sequence (5' - 3')
**SGyA-1F**	AACAGTATATTTGTGGTGTGCCAACCAACAACGGCGCGCCTGCAGCTGGTTGTA
**SGyA-1R**	AATTGAATTAGATCCCCGGGCGAGCTCGAATTGCTAGCCGGCCGTTAACTCGAAT
**SGyA-2F**	AATTGAATTAGATCCCCGGGCGAGCTCGAATTGCTAGCCGGCCGTTAACTCGAAT
**1054A.1**	TTTGCTGAGTCGTTCTCCTATTACATAGTCCG
**842B**	ATCAACGATGCCCAGCTGGC
**1054A.3**	AGTTGTGGTTTGTCCAAACTCATCAATGTA
**1054A.2**	CTGAACATAAGATCCAACCATGCTTC
**1054A.5**	CAGGATCTTGTGGTGCTAAGTCAGATGCAGT
**1054A.6**	GGAGGAAAGTTTCGGTCAGAAGCCTC
**1054A.7**	GTGGGTTCCTGTGGTCTTCC
**1054A.8**	GTTGCCGAGCACAATTGATC
**640B**	CTTCACGTTTTCCCAGGTCAGAA
**941G.SQ2**	CAGTCAGTCAAAGCAGACAACAAATGAG
**SGyA gRNA-F**	GAAATTAATACGACTCACTATAGGTTATTTGGCACTACTTCTGTTTTAGAGCTAGAAATAGC
**SGyA gRNA-R**	AAAAGCACCGACTCGGTGCCACTTTTTCAAGTTGATAACGGACTAGCCTTATTTTAACTTGCTATTTCTAGCTCTAAAAC
**GDe.1F**	GCTTCTGGATGGCTGTGATCATG

**GDe.10R**	AGCTCGCTATACACTCGCATG
**GDe.9F**	GTCTATATCATGGCCGACAAGC
**GDe.2R**	CTCTCCCAGATCTCAACTTTGG
**GDe.3R**	CGGTACATCGCTTATGTGTATG
**se-F**	GAGCAATATGCAGGTTGCAAGG
**se-R**	GCACCTAACACCGCTCTAAATC
**e-F**	CACGCCCTCATCGAAATAGTC
**e-R**	GATTCGCGACGATGACATCGAC
**f-F**	CGCAAATGGACAACGATGTCACG
**f-R**	GGCCAATGCACTTAACGTAGC
**cu-F**	CAACAATGTGGACTCCCAGGACGATG
**cu-R**	CTACCCAACACGGTCTGCAGGAACTTG
**Dmelwg761.1**	CTATAACTAGAGCGAGCCGA
**Dmelwg761.2**	GATCCCCAAGTCTTGACTTC
**Dmelwg761.3**	GTGCGCTAATTGAACGCGAA
**Dmelwg761.4**	GTGTGCATTTTGTGGCTTCC
**Dmelcut767.1**	CAACAAACGACATCGAGGAC
**Dmelcut767.2**	GCCGCCATTGTTGTTCTTTC
**Dmelcut767.3**	GCCTCAATCCCAAGCTTTTC
**Dmelcut767.4**	CTTGGCTCAGTTCTGGTTTC
**DmelAp769.1**	GAGGTTGCGCGTTATTTTGC
**DmelAp769.2**	CTAATGATCACACCACCCAC
**DmelAp769.3**	TCCTCAAAGGTAGCACGATC
**DmelAp769.4**	CTCTCCTCGAAGAGCTTTCT
**DmelTw773.1**	GTGTGCACCACTCTAAGAAG
**DmelTw773.2**	GAATCCCATGGGCATGATCA
**DmelTw773.3**	TGTTAAAACCCCCAAGTGCC
**DmelTw773.4**	GTTGTACTTGTCACCTGGCTTC
**DmelSc774.1**	CTTTCCAATGGGCAGAGAAG
**DmelSc774.2**	CAGCGGATGACAAGTCCTTT
**DmelSc774.3**	CAGATCTGAGATCCTTTTGGG
**DmelSc774.4**	GAGTTTTTGGCGTACAGTGC
**gRNA *forked***	TTGTACGTCCGTGCACGCGA

**gRNA *ebony***	ATCGAGTCCACGAAGGTTA
**gRNA *curled***	ATGACATTCGATTGCTGCAG
**gRNA sepia**	ATTTGCCCAACGGGTGCATC
**gRNA *apterous***	TTGATGTGCTACAGGTGGTG
**gRNA *scalloped***	GATAGCAAAAACCTGGATGT
**gRNA *cut***	CTCCATTCGCCGGCGAATGA
**gRNA *wingless***	GAAGGGGCCGGGGCTCCATG
**gRNA *twisted***	CTTCTATAAGGTCACCGAGC
