# Supplementary material for: Exploiting a Y chromosome-linked Cas9 for sex selection and gene drive
Source: Nat Commun. 2021 Dec 10;12:7202. doi: 10.1038/s41467-021-27333-1 (PMC8664916; doi:10.1038/s41467-021-27333-1)
Supplement: Supplementary file 2 — Reporting Summary [file 41467_2021_27333_MOESM2_ESM.pdf]

nature portfolio

Corresponding author(s): Omar S. Akbari

Last updated by author(s): 2021/10/26

# Reporting Summary

Nature Portfolio wishes to improve the reproducibility of the work that we publish. This form provides structure for consistency and transparency in reporting. For further information on Nature Portfolio policies, see our Editorial Policies and the Editorial Policy Checklist.

## Statistics

For all statistical analyses, confirm that the following items are present in the figure legend, table legend, main text, or Methods section.

| n/a | Confirmed | |
|---|---|---|
| ☐ | ☒ | The exact sample size (*n*) for each experimental group/condition, given as a discrete number and unit of measurement |
| ☐ | ☒ | A statement on whether measurements were taken from distinct samples or whether the same sample was measured repeatedly |
| ☐ | ☒ | The statistical test(s) used AND whether they are one- or two-sided<br>*Only common tests should be described solely by name; describe more complex techniques in the Methods section.* |
| ☒ | ☐ | A description of all covariates tested |
| ☐ | ☒ | A description of any assumptions or corrections, such as tests of normality and adjustment for multiple comparisons |
| ☐ | ☒ | A full description of the statistical parameters including central tendency (e.g. means) or other basic estimates (e.g. regression coefficient) AND variation (e.g. standard deviation) or associated estimates of uncertainty (e.g. confidence intervals) |
| ☐ | ☒ | For null hypothesis testing, the test statistic (e.g. *F*, *t*, *r*) with confidence intervals, effect sizes, degrees of freedom and *P* value noted<br>*Give P values as exact values whenever suitable.* |
| ☒ | ☐ | For Bayesian analysis, information on the choice of priors and Markov chain Monte Carlo settings |
| ☒ | ☐ | For hierarchical and complex designs, identification of the appropriate level for tests and full reporting of outcomes |
| ☒ | ☐ | Estimates of effect sizes (e.g. Cohen's *d*, Pearson's *r*), indicating how they were calculated |

*Our web collection on statistics for biologists contains articles on many of the points above.*

## Software and code

Policy information about availability of computer code

| | |
|---|---|
| Data collection | No computer software was used for data collection. For the modelling section of the paper, simulations were performed using the MGDrivE package (version 1.6.0) in R (https://www.r-project.org/) and analyzed using the MoNeT Python package (https://pypi.org/project/MoNeT-MGDrivE) |
| Data analysis | The software used for analyses was GraphPad Prism version 8.3.1 for macOS (GraphPad Software, San Diego). To analyze RNA-seq data, reads were mapped to the Drosophila melanogaster genome (Dmel release 6) using STAR aligner 71, and the expression levels were determined with featureCounts 72. Correlation coefficients of the transcripts-per-million (TPM) values between WT and transgenic animals were calculated in R[14] and plotted with ggplot2. Differential expression analysis between transgenic and WT samples was performed using DESeq2. GOstats R software package was used to determine overrepresented GO terms. |

For manuscripts utilizing custom algorithms or software that are central to the research but not yet described in published literature, software must be made available to editors and reviewers. We strongly encourage code deposition in a community repository (e.g. GitHub). See the Nature Portfolio guidelines for submitting code & software for further information.

## Data

Policy information about availability of data

All manuscripts must include a data availability statement. This statement should provide the following information, where applicable:
- Accession codes, unique identifiers, or web links for publicly available datasets
- A description of any restrictions on data availability
- For clinical datasets or third party data, please ensure that the statement adheres to our policy

Complete sequence and plasmid DNA for vector SGyA is available at Addgene (#160292). The data generated in this study are provided in the Supplementary

Information/Source Data file. All RNA sequencing data are available for download at NCBI with BioProject: PRJNA748400 [https://dataview.ncbi.nlm.nih.gov/object/PRJNA748400?reviewer=t07d7rithq0vq9dgm68468okmc].

# Field-specific reporting

Please select the one below that is the best fit for your research. If you are not sure, read the appropriate sections before making your selection.

[x] Life sciences [ ] Behavioural & social sciences [ ] Ecological, evolutionary & environmental sciences

For a reference copy of the document with all sections, see nature.com/documents/nr-reporting-summary-flat.pdf

# Life sciences study design

All studies must disclose on these points even when the disclosure is negative.

| | |
|---|---|
| Sample size | At least 3 biological replicates were performed per experiment. No sample size calculation was performed. Sample sizes were chosen according to the scientific literature of the field. All raw data is presented in the Source data file. |
| Data exclusions | For embryo and adult counts (to determine hatching rate): data was omitted for crosses that had adult counts below 15. This was done to prevent low numbers from skewing the data. Low embryo counts often corresponded with fertility rates and often corresponded to sick/ low fecundity flies that are not useful for the experiment. Thus, only crosses which had high fecundity and fertility were kept for analysis. |
| Replication | Each experimental cross was repeated at least 3 times using 10 females (WT, gRNA, or GDe) and 10 males (WT, autosomal-, Y-, X-, linked Cas9) per cross. Total numbers of flies counted/scored are indicated in the Source Data file for each respective experiment/figure. |
| Randomization | All experimental crosses were set up using randomly sampled flies. |
| Blinding | All flies were blindly chosen at random for all experimental crosses, molecular work, and imaging. |

# Reporting for specific materials, systems and methods

We require information from authors about some types of materials, experimental systems and methods used in many studies. Here, indicate whether each material, system or method listed is relevant to your study. If you are not sure if a list item applies to your research, read the appropriate section before selecting a response.

## Materials & experimental systems

| n/a | Involved in the study | |
|---|---|---|
| [x] | [ ] | Antibodies |
| [x] | [ ] | Eukaryotic cell lines |
| [x] | [ ] | Palaeontology and archaeology |
| [ ] | [x] | Animals and other organisms |
| [x] | [ ] | Human research participants |
| [x] | [ ] | Clinical data |
| [x] | [ ] | Dual use research of concern |

## Methods

| n/a | Involved in the study | |
|---|---|---|
| [x] | [ ] | ChIP-seq |
| [x] | [ ] | Flow cytometry |
| [x] | [ ] | MRI-based neuroimaging |

## Animals and other organisms

Policy information about studies involving animals; ARRIVE guidelines recommended for reporting animal research

| | |
|---|---|
| Laboratory animals | Drosophila melanogaster W[1118] (male and female stock; estimated 5 years old), a transgenic gRNA line (U6:3-t::gRNA-se:e:cu:f) from a previously published paper PMCID: PMC5215823 (male and female stock; estimated 3 years old), transgenic lines from Bloomington Stock Center expressing sgRNAs targeting the genes wingless (BDSC# 81980), cut (BDSC#81942), apterous (BDSC#80345), twisted (BDSC#76991) and scalloped (BDSC#77055), an autosomal linked vasa-Cas9 (BDSC#79006) and the transgenic SGyA line that was established in this study (BDSC#91386). BDSC sgRNA stocks consisting of males and females, are estimated to be 2 years old. SGyA line is composed of transgenic males and non-transgenic females---approximately 3 years old. |
| Wild animals | No wild animals |
| Field-collected samples | No field collected samples |
| Ethics oversight | We have complied with all relevant ethical regulations for animal testing and research and conformed to the UCSD institutionally approved biological use authorization protocol (BUA #R2401). |

Note that full information on the approval of the study protocol must also be provided in the manuscript.
