# Supplementary material for: Exploiting a Y chromosome-linked Cas9 for sex selection and gene drive
Source: Nat Commun. 2021 Dec 10;12:7202. doi: 10.1038/s41467-021-27333-1 (PMC8664916; doi:10.1038/s41467-021-27333-1)
Supplement: Supplementary file 4 — Description of Additional Supplementary Files [file 41467_2021_27333_MOESM4_ESM.pdf]

Title: Supplementary Data File 1.
Description: Quantification of total RNA expression in WT, autosomal-Cas9, and SGyA adult ♂ whole body at 3-4 days old. Both raw read counts and normalized TPM values are included.

Title: Supplementary Data File 2.
Description: Two-factor comparison between autosomal Cas9 and WT.

Title: Supplementary Data File 3.
Description: Two-factor comparison between SGyA and WT.

Title: Supplementary Data File 4.
Description: List of model and intervention parameters.
